# Changes in cannabis use post decriminalisation in mental health care users in South Africa

**DOI:** 10.4102/sajpsychiatry.v31i0.2305

**Published:** 2025-03-10

**Authors:** Rachel Moshori, Lisa Galvin, Laila Paruk

**Affiliations:** 1 Department of Psychiatry, Faculty of Health, University of the Witwatersrand, Johannesburg, South Africa

**Keywords:** cannabis, decriminalisation, psychotic disorders, South Africa, mental health, substance use, psychiatric admissions

## Abstract

**Background:**

Personal cannabis use was decriminalised in South Africa in 2018. Cannabis use increases the risk of mental illness (MI) and worsens prognosis in patients with MI. The impact of decriminalisation on cannabis use remains unclear.

**Aim:**

To examine cannabis use patterns by self-report and urine multidrug screening (UMDS) among psychiatric inpatients at Chris Hani Baragwanath Academic Hospital (CHBAH).

**Setting:**

CHBAH, Soweto, South Africa.

**Methods:**

A retrospective review of clinical records comparing patients admitted to CHBAH psychiatry pre- and post-decriminalisation.

**Results:**

A total of 244 patients were included, with 57% using cannabis based on self-report and/or UMDS. Although not significant, overall cannabis use was higher post-decriminalisation (63.1%) than pre-decriminalisation (50.8%). Self-reported use increased slightly post-decriminalisation (56.6% vs. 50.0%), while UMDS-confirmed use was significantly higher (32.8% vs. 17.2%). Cannabis use was associated with male gender and younger age, with younger admissions post-decriminalisation.

**Conclusion:**

Decriminalisation did not significantly increase overall or self-reported cannabis use; however, more patients tested positive on UMDS post-decriminalisation. Young males remain at higher risk of cannabis use.

## Introduction

Cannabis is the most widely used recreational substance globally, with approximately 200 million users, and is the most used drug in South Africa.^1,2^ In South African substance rehabilitation facilities, 43% of patients tested positive for cannabis.^3^ Several factors contribute to this, including male gender, unemployment, being single, recurring hospitalisations and low socioeconomic status.^2,3,4^ Mental health care users (MHCUs) frequently use cannabis because it is widely available, seen as socially acceptable and often perceived as harmless.^5^

Cannabis use has been reported in up to 50% of inpatient MHCUs in South Africa.^6^ Cannabis was the most used drug among MHCUs with comorbid human immunodeficiency virus (HIV) infection attending outpatient psychiatric services at Chris Hani Baragwanath Academic Hospital (CHBAH) in Soweto, with 29% of virologically suppressed, and 46.1% of unsuppressed HIV-infected MHCUs using cannabis.^7^ In KwaZulu-Natal, MHCUs were found to be at an 11-fold increased risk of testing positive for cannabis compared to general medical patients.^3^ In contrast, two-thirds of adolescent MHCUs in the same region reported cannabis use.^8^

In 2018, South Africa’s Constitutional Court decriminalised the personal use of cannabis.^9^ Supporters of this policy emphasise the potential medicinal benefits of cannabis, including treating pain, chemotherapy-related nausea, spasticity in multiple sclerosis, social anxiety disorder and short-term sleep disturbances.^10,11^ Additionally, decriminalisation could ease the burden on the criminal justice system, reduce organised crime, create a regulatory framework for cannabis products, generate revenue to boost the economy and allow expanded research on its medical applications.^12,13,14,15^ Proponents also argue that decriminalisation may empower patients with cannabis use disorders (CUDs) to disclose their use without fear of stigma or legal consequences, potentially improving their access to healthcare.^15,16^

However, there are arguments against decriminalising cannabis. Despite potential benefits, use has been linked to significant medical and mental health concerns. These include an increased risk of respiratory and cardiovascular diseases and a higher likelihood of traumatic injuries.^17,18^ A systematic review of the literature examining cannabis use following its legalisation in Canada and the United States reported increases in cannabis-related healthcare visits, impaired driving and emergency room admissions.^19^ The impact of prenatal cannabis exposure on offspring behaviour and cognitive development remains contentious, although possible adverse effects, such as low birth weight, have been reported.^20^ Cannabis use among adolescents and young adults may impair brain development, with studies highlighting reduced brain volume in critical areas such as the hippocampus and prefrontal cortex.^21^ In the context of HIV and tuberculosis, cannabis use has been associated with poor medication adherence and worse health outcomes, exacerbating South Africa’s burden of disease.^22,23^

Cannabis has also been linked to adverse mental health outcomes such as younger onset of MI, prolonged duration of untreated psychosis, higher relapse rates, treatment non-adherence and increased hospitalisation rates among MHCUs.^24,25,26,27,28^

Proponents of cannabis decriminalisation suggest it may improve access to healthcare. Conversely, it could reduce patients’ motivation to access healthcare treatment for CUDs because of lowered perception of the associated risks.^19,29^

Globally, the impact of cannabis decriminalisation has been mixed. In some countries, decriminalisation has led to increased use. Following legalisation in the USA and Canada, several studies reported a rise in use, particularly among adults.^19,30^ However, data from Portugal, which decriminalised all drugs in 2001, indicate that adolescent drug use, including cannabis, has decreased.^31^

In South Africa, most studies on cannabis use predate decriminalisation.^3,8^ A recent study in Cape Town found that use in adolescents admitted with MI increased after the decriminalisation.^32^ The impact of decriminalisation of cannabis use in adult MHCUs in South Africa remains unknown.

Urine multidrug screening (UMDS) is frequently employed to supplement clinical history and facilitate early detection and intervention in substance use disorders.^33^ Cannabis can be detected in urine for up to 30 days, depending on usage patterns.^33^ While self-reported cannabis use is often consistent with UMDS results, it is unclear whether UMDS testing offers benefits beyond self-reporting.^34^ Rarely, false positives can occur because of drug cross-reactivity, such as with efavirenz or ibuprofen.^35^ False positives may have significant consequences, including patients being denied access to rehabilitation programmes, employment termination or negative bias from medical staff.^36^

Knowledge is limited about the impact of decriminalisation on cannabis use among South African MHCUs. To the best of our knowledge, this study is the first to explore cannabis use in a South African acute adult psychiatric admissions unit since decriminalisation.

### Aim

The study aims to examine patterns of cannabis use among MHCUs admitted to the acute admission ward at CHBAH before and after decriminalisation.

The study examined cannabis use on UMDS and self-reported cannabis use.

## Research methods and design

### Study design

A descriptive observational analysis with a pre- and post-design using data collected from a retrospective record review.

### Setting

The psychiatry admission ward of CHBAH, a tertiary-level hospital servicing Soweto, a peri-urban area in South Africa.

### Study population and sampling strategy

Patients 18 years and older admitted to the acute psychiatric ward from 31 March 2017 to 31 March 2018 (pre-decriminalisation) and 31 March 2019 to 31 March 2020 (post-decriminalisation) were included. Psychiatric interviews and UMDS are conducted on all patients at admission. Patients under 18 or with missing files or incomplete notes and no substance history documented during admission were excluded. Patients were included if a substance history was documented, even if the UMDS test result was missing.

If patients had multiple admissions, only the first ones in the pre- and post-decriminalisation 12 months were captured. Subsequent admissions were excluded.

We employed a systematic sampling method to select patient files. Every fourth file from patients admitted during the study timeframe was selected from the admissions register. The files corresponding to these numbers were accessed from the hospital registry. This approach was used to ensure a representative sample and minimise selection bias. By consistently selecting every fourth file, we aimed to avoid over-representing specific admission periods or types of patients.

The sample size was determined using *n* = (Zα/2+Zβ)2 * p1(1-p1) + p2(1-p2) / (p1-p2)2, where Zα/2 and Zβ are the z-statistics, and p1, p2 are the two proportions before and after decriminalisation of cannabis. With 80% power, a two-sided test, significance level α = 0.05 and an assumption of p1 = 0.25 and p2 = 0.45, it was estimated that 256 participants would be enrolled with an equal split between the periods before and after decriminalisation, that is 128 per group.

### Data collection

Data were collected from the records accessed from the registry, starting with the pre-decriminalisation cohort. Six records were missing from the pre-decriminalisation group. The same process was used for the post-decriminalisation cohort until an equal number of records were obtained for both cohorts to ensure comparability. Demographics, clinical information and substance history were gathered from the admissions records of the Department of Psychiatry at CHBAH. Data from clinical records included gender, age, relationship status, highest level of education and employment status. Presenting symptoms were collected rather than a definitive diagnosis as information was obtained from initial history, and a diagnosis may not have been made yet. The literature discusses psychotic and mood disorders in broad terms rather than focusing on discrete diagnoses, and assessing these broad groups improved comparability to pre-existing literature.^4^ Psychiatric admission history, route of admission, UMDS results and self-reported substance use were recorded. Chris Hani Baragwanath Academic Hospital uses a qualitative lateral flow immunochromatographic urinary assay multidrug five-panel test for cocaine, methamphetamine, amphetamine, cannabis and opioids. This bedside test does not require laboratory investigation, thus reducing the probability of a lost result. Psychiatric nursing staff experienced in administering and interpreting the test administered it on admission. Data were captured on the Research Electronic Data Capture (REDCAP) database.^37^

### Data analysis

The SAS Enterprise Guide 7.1 methods of frequency analysis, mean analysis, nonparametric one-way analysis, *t*-test and generalised linear model were used for statistical analysis. The categorical data analysis resulted in frequencies and percentages stratified by the decriminalisation of cannabis (i.e. pre- vs. post-decriminalisation). Fisher’s or the Chi-Square test analysis was employed to determine statistical significance for categorical measures stratified by cannabis decriminalisation. Kruskal–Wallis’s test and Student’s *t*-test were used to compare continuous measures non-parametrically and parametrically. Continuous measures were assessed using medians, interquartile ranges (IQR), means and standard deviations.

Lifetime use was defined as any self-reported use of cannabis at any time in the patient’s life.

Ongoing use was defined as self-reported cannabis use or a cannabis-positive UMDS result at the time of consultation. Patients were defined as not having ongoing use if self-reported cannabis use had not occurred within the past 30 days and there was no cannabis-positive UMDS result at the time of consultation.

### Ethical considerations

Ethical clearance to conduct this study was obtained from the University of the Witwatersrand Human Research Ethics Committee (No. M221005 MED-09-067). There was no risk to patients as this was a retrospective record review with no patient identifiers. Only anonymised patient data were used, ensuring patients’ identities remain confidential. Informed consent was, therefore, not required.

## Results

### Demographic characteristics

The total study population was 246 patients. After excluding two patients with missing data (one from the pre- and one from the post-decriminalisation group), 244 patients were included in the final analysis, with an equal distribution of 122 patients in each group. There were also 12 fewer files (six in both the pre- and post-decriminalisation arm) captured than anticipated because of entire files not being traced. Most patients were male (60.7%; *n* = 148/244), 18–35 years old (52.5%; *n* = 128/244), single (82.4%; *n* = 201/244) and unemployed (87.3%; *n* = 213/244) (Table 1). Patients in the post-decriminalisation cohort were younger (33 vs. 36 years; *p* = 0.0194) than pre-decriminalisation (Table 1).

**TABLE 1:** Demographic characteristics by cannabis decriminalisation.

Variable	Overall				Pre-decriminalisation				Post-decriminalisation				*P*
	*N* = 244				*N* = 122				*N* = 122				
	*n*	%	Median	IQR	*n*	%	Median	IQR	*n*	%	Median	IQR	
**Gender**													
Male	148	60.7	-	-	80	65.6	-	-	68	55.7	-	-	0.1158
Female	96	39.3	-	-	42	34.4	-	-	54	44.3	-	-	-
**Age (years)**													
18–35	128	52.5	-	-	56	45.9	-	-	72	59.0	-	-	**0.0403**
36–48	70	28.7	-	-	40	32.8	-	-	30	24.6	-	-	0.1570
49–61	29	11.9	-	-	17	13.9	-	-	12	9.8	-	-	0.3226
> 61	17	7.0	-	-	9	7.4	-	-	8	6.6	-	-	0.8015
	24	-	435.0	28.0–44	12	-	236.0	30.0–46	12	-	233.0	27.0–43	0.0194
**Relationship status**													
In a relationship	43	17.6	-	-	26	21.3	-	-	17	13.9	-	-	0.1305
Not in a relationship	201	82.4	-	-	96	78.7	-	-	105	86.1	-	-	-
**Highest level of education**													
None and/or unknown	37	15.2	-	-	19	15.6	-	-	18	14.8	-	-	0.8932
Grade 1–8	31	12.7	-	-	16	13.1	-	-	15	12.3	-	-	-
Grade 9–12	153	62.7	-	-	74	60.7	-	-	79	64.8	-	-	-
Post matric	23	9.4	-	-	13	10.7	-	-	10	8.2	-	-	-
**Employment Status**													
Employed	29	11.9	-	-	18	14.8	-	-	11	9.0	-	-	0.1490
Unemployed	213	87.3	-	-	104	85.2	-	-	109	89.3	-	-	-
Unknown	2	0.8	-	-	0	0.0	-	-	2	1.6	-	-	-

IQR, interquartile ranges.

### Clinical characteristics

The commonest presenting symptom was psychosis (47.5%; *n* = 116), followed by 29.1% (*n* = 71) presenting with mood and psychotic symptoms and 9.0% (*n* = 22) presenting with mood symptoms only. Intoxication was recorded in 4.9% (*n* = 12) of patients. ‘Other’ was recorded in 9.4% (*n* = 23) of files, consisting of intellectual disability (*n* = 3/27) and neurocognitive (*n* = 8/27), personality (*n* = 8/27), panic (*n* = 1/27), post-traumatic stress (*n* = 1/27) and adjustment disorders (*n* = 2/27). There were no significant associations between decriminalisation and presenting symptoms. Index presentation was noted for 34.4% (*n* = 84/244) of patients with no association with decriminalisation (*p* = 0.1779) (Table 2).

**TABLE 2:** Clinical characteristics by cannabis decriminalisation.

Variable	Overall		Pre-decriminalisation		Post-decriminalisation		*P*
	*N* = 244		*N* = 122		*N* = 122		
	*n*	%	*n*	%	*n*	%	
**Symptoms**							
Psychosis	116	47.50	58	47.50	58	47.54	0.4264
Mood	22	9.0	13	10.70	9	7.38	
Psychosis + Mood	71	29.1	30	24.60	41	33.61	
Substance intoxication	12	4.92	7	5.74	5	4.10	
Other	23	9.4	14	11.50	9	7.38	
**Previous psychiatric admission**							
Index presentation to psychiatry	84	34.43	47	38.52	37	30.33	0.1779
Known mental health care user	160	65.57	75	61.48	85	69.67	
**Route of access to care**							
Self-referred	13	5.33	8	6.56	5	4.10	0.3925
Family	133	54.51	55	45.08	78	63.93	**0.0031**
SAPS (police)	25	10.25	7	5.74	18	14.75	**0.0202**
EMS (emergency medical services)	73	29.92	52	42.62	21	17.21	**< 0.0001**
**Route of admission (Where admitted from)**							
MEU (casualty)	162	66.39	60	49.18	102	83.61	**< 0.0001**
Specialist clinic	5	2.05	5	4.10	0	0.00	-
Psychiatry OPD	10	4.10	7	5.74	3	2.46	0.1965
Another ward	18	7.38	12	9.84	6	4.92	0.1417
Other clinic or Hospital	49	20.08	38	31.15	11	9.02	**< 0.0001**

Note: Figures in bold highlights statistical significance (*p* ≤ 0.05).

SAPS, South African Police Service; MEU, Medical Emergency Unit; OPD, Outpatient Department.

Over half of the patients (54.5%; *n* = 133/244) were brought to the hospital by family, followed by emergency medical services (EMS) (29.9%; *n* = 73/244) and police (10.2%; *n* = 25/244). Most patients, 66.4% (*n* = 162/244), were admitted from the medical emergency unit (MEU). Fewer patients, 17.2% (*n* = 21/122) (*p* < 0.0001), arrived by EMS post- versus pre-decriminalisation (42.6%; *n* = 52/122). Conversely, more patients, 63.9% (*n* = 78/122), were brought by family post- compared to pre-decriminalisation (45.1%; *n* = 55/122; *p* = 0.0031). More patients (83.6%; *n* = 102/122) were admitted via MEU post- than pre-decriminalisation (49.2%; *n* = 60/122; *p* < 0.0001) (Table 2).

### Substance use

A total of 57.0% of the patients (*n* = 139/244) reported lifetime cannabis use, either confirmed by self-report or using UMDS. There was no statistically significant difference in lifetime cannabis use pre- (50.8%; *n* = 62/122) compared to post-decriminalisation (63.1%; *n* = 77/122; *p* = 0.0524) (Table 3).

**TABLE 3:** Substance use by cannabis decriminalisation.

Variable	Overall		Pre-decriminalisation		Post-decriminalisation		*P*
	*N* = 244		*N* = 122		*N* = 122		
	Overall		Yes (*n* = 61)		No (*n* = 183)		
	*n*	%	*n*	%	*n*	%	
**Age group and cannabis use**							
18–35	128	52.2	43	70.5	85	46.5	**0.0011**
36–48	70	28.7	12	19.7	58	31.7	0.0722
49–61	29	11.9	5	8.2	24	13.1	0.3040
> 61	17	7.0	1	1.6	16	8.7	0.0591
**All lifetime cannabis use**							
No	105	43.0	60	49.2	45	36.9	0.0524
Yes (%) Lifetime cannabis use	139	57.0	62	50.8	77	63.1	
**Urine multidrug screening result**							
Cannabis	61	25.0	21	17.2	40	32.8	**0.0050**
Not done	108	44.3	66	54.1	42	34.4	**0.0020**
Negative for all substances	51	20.9	23	18.9	28	23.0	0.4311
Unknown*	2/244		0/244		2/244		-
**Self-reported substance use (lifetime use)**							
Cannabis	130	53.3	61	50.0	69	56.6	0.3047
Methamphetamine	26	10.7	12	9.8	14	11.5	0.6782
Opioids	14	5.7	7	5.7	7	5.7	0.9999
Other	15	6.1	9	7.4	6	4.9	0.4240
None	102	41.8	54	44.3	48	39.3	0.4361
**Ongoing self-reported cannabis use**							
Previous cannabis use	26/130	20.0	17/61	27.9	9/69	13.0	**0.0350**
Ongoing cannabis	104/130	80.0	44/61	72.1	60/69	87.0	
**Self-reported cannabis use with positive cannabis urine drug test (lifetime use)**							
No	9/61	14.8	1/21	4.8	8/40	20.0	0.1108
Yes	52/61	85.2	20/21	95.2	32/40	80.0	

Note: Figures in bold highlights statistical significance (*p* ≤ 0.05).

* , Two patients underwent UMDS testing, but their results were not documented and were recorded as ‘unknown’. Because of the small sample size, no statistical analysis was conducted on these cases.

No UMDS was done for 44.3% (*n* = 108/244) of participants. Urine multidrug screening was positive for cannabis in 25.0% (*n* = 61/244) of patients. Only 20.9% (*n* = 51/244) of patients had a negative UMDS result. Two UMDSs were documented as done, but the results were not recorded. Results on UMDS also included methamphetamines, 5.3% (*n* = 13/244) and ‘other’. Within the ‘other’ group, six patients tested positive for benzodiazepines, two for opioids and one for tricyclic antidepressants.

There was no statistical difference in negative UMDS results in the pre- versus post-decriminalisation groups (18.9%; *n* = 23/122 vs. 23.0%; *n* = 28/122; *p* = 0.4311). More patients (32.8%; *n* = 40/122) tested positive for cannabis post- compared to pre-decriminalisation (17.2%; *n* = 21/122; *p* = 0.0050). When examining the number of positive cannabis results in only patients who had a documented UMDS result, 45.5% (*n* = 61) of them tested positive for cannabis. In the group with documented UMDS results, a higher percentage tested positive for cannabis post- compared to pre-decriminalisation (55.5%; *n* = 40/72 vs. 38.6%; *n* = 21/56), with this difference reaching statistical significance (*p* = 0.05).

Younger patients were more likely to have a cannabis-positive UMDS result, with 70.5% (*n* = 43/61) being in the 18–35 age group compared to 46.5% (*n* = 85/183) who did not test positive for cannabis (*p* = 0.0011) (Table 3).

A total of 53.3% (*n* = 130/244) of patients self-reported lifetime cannabis use with no statistically significant difference between pre- and post-decriminalisation groups (50.0%; *n* = 61/122 vs. 56.6%; *n* = 69/122; *p* = 0.30467). Of the 130 patients reporting lifetime cannabis use, 80.0% (*n* = 104/130) reported ongoing use. Of those with ongoing cannabis use, 90.4% (*n* = 94/104) reported having used cannabis ≤ 30 days before admission, and 9.6% (*n* = 10/104) had not used cannabis in over 30 days before admission. More patients reported current cannabis use post- compared to pre-decriminalisation (87.0%; *n* = 60/69 vs. 72.1%; *n* = 44/61; *p* = 0.035) (Table 3).

Of the 61 patients who tested positive for cannabis on UMDS, 85.2% (*n* = 52/61) had self-reported cannabis use, while 14.8% (*n* = 9/61) had not disclosed use. In the pre-decriminalisation group, 4.8% (*n* = 1/21) with a positive UMDS did not disclose cannabis use compared to 20.0% (*n* = 8/40) post-decriminalisation (*p* = 0.1108) (Table 3).

## Discussion

Lifetime cannabis use was prevalent in more than half the patients in our cohort, confirmed by UMDS and/or self-reporting. Lifetime cannabis use increased post-decriminalisation, although only cannabis use by UMDS result was statistically significant. Young males made up most of the admissions, which remained unchanged after decriminalisation.

Our results were in keeping with other studies, which report that roughly half of South African MHCUs used cannabis.^6,24,31^ Overall, cannabis use in our cohort increased by 12% post-decriminalisation. Cannabis use by UMDS results almost doubled post-decriminalisation. However, this result was inflated by the number of missing UMDS results. When only examining patients with a documented UMDS result, however, there was still a 17% increase in cannabis use post-decriminalisation. Despite the lack of statistical significance for the increase in lifetime cannabis use by self-report and cannabis use in general, the trends for an increase in ongoing cannabis use are supported by UMDS results. These results support findings in Cape Town of increased cannabis use among adolescents admitted with MI post-decriminalisation.^32^

This study supports international literature that has observed increased cannabis use post-legalisation in regions such as the USA and Canada.^30,38,39^ Studies in these countries have shown a rise in cannabis use among adults after the decriminalisation of personal cannabis use.^30,38^ However, Farrelly et al. found no significant increase after legalisation in the US, reflecting the mixed outcomes in research.^19^ These differences may be because of varying study methodologies, population demographics and definitions of cannabis use across regions.^40^ Furthermore, the year of cannabis legalisation and societal shifts, such as changes in perceptions of cannabis use, may contribute to these mixed findings.^30,40^

Among those who self-reported cannabis use, a higher percentage in the post-decriminalisation group (87.0%; *n* = 60) reported ongoing use, compared to 72.1% in the pre-decriminalisation group (Table 3). A possible explanation for the increase in current ongoing self-reported cannabis use is that patients may feel more comfortable reporting use post-decriminalisation as they would not face legal consequences. However, we were not able to support this conclusion. Although self-reported use did increase after decriminalisation, it was not large enough to be statistically significant. This suggests that decriminalisation has not impacted patient willingness to self-disclose. Despite the missing UMDS data, the significant increase in positive cannabis tests post-decriminalisation (55.5% vs. 38.6%) suggests a genuine rise in cannabis use, indicating that missing results do not fully account for the observed difference. It is possible that it was too soon after decriminalisation for changes to occur in how patients view cannabis (e.g. self-stigma). Possible barriers to self-disclosure may still exist, such as social desirability and fears of confidentiality breach.^41,42,43^

Of the 61 patients with a positive cannabis UMDS result, 85.2% (*n* = 52) self-disclosed cannabis use and a further 14.8% (*n* = 9) using cannabis were identified by UMDS result alone. International literature reports similar findings, with 19% of cannabis users in a Persian cohort study being detected by UMDS despite denying use.^44^

Urine multidrug screening may offer objective information on recent drug use and potentially provide therapeutic benefits by encouraging abstinence; however, there is limited evidence around UMDS as a therapeutic tool in changing patient behaviour.^45^ It is uncertain from our results if there is value to administering a UMDS, as self-reported cannabis use constituted 53% of the total sample. In comparison, UMDS results only identified cannabis use in 45.5% of those patients with documented cannabis-positive results. The additional identification by UMDS of a further 14.8% of patients using cannabis who had not self-disclosed conversely suggests there may be possible value in detecting patients using cannabis who do not self-disclose. It is difficult to assess if these results are genuinely patients who do not disclose or if some may be false positives. More research is necessary to understand resource implications and cost-effectiveness of UMDS use post-decriminalisation. Farrelly et al. similarly found discrepancies between self-reported cannabis use and objective testing, highlighting the importance of urine screening in accurately detecting cannabis use, which aligns with our findings.^19^

### Demographic characteristics

Almost two-thirds of the cohort were male, with no significant differences observed between the pre- and post-decriminalisation groups. The most common age group was 18–35 years, and a notable increase in admissions in this age bracket was observed following decriminalisation (Table 1). This is in keeping with the literature, which demonstrates that being male is a risk factor for developing MI and using cannabis.^6,46,47,48,49^ The fact that two-thirds of the sample were male, and half the sample were under the age of 35 years emphasises that young males who use cannabis make up a substantial percentage of admitted MHCUs. Given that cannabis use is associated with poor mental health outcomes, such as earlier age of psychosis onset and treatment non-adherence, it is unsurprising that young males account for most admissions in our study.^50,51^ Decriminalisation does not appear to have changed the fact that males are more likely than females to be admitted with MI. Our study was focused on psychiatric admissions, not the overall prevalence of MI. It showed that males consistently remain at higher risk for psychiatric admissions related to cannabis use across pre- and post-decriminalisation periods.^32^ Although the proportion of male admissions did not change between the two periods, there was a shift towards admitting younger patients in the post-decriminalisation period.

It is also important to note that admissions are not synonymous with MI prevalence. While males may dominate in psychiatric admissions, this does not necessarily reflect higher rates of MI among men. Factors such as societal roles, child-rearing responsibilities or earlier help-seeking behaviour may result in women being more likely to seek outpatient care rather than hospital admission.^48^ Therefore, the admission rates observed in this study may reflect the severity of illness or the nature of care pathways rather than the true prevalence of MI across genders.

Psychiatric admission can indicate severe MI, as outpatient care is preferred because of resource constraints.^52^ Younger age at admission post-decriminalisation may suggest earlier development of severe MI, given cannabis’s link to poor mental health outcomes such as earlier onset of psychosis.^26,27,52^

Reduction of stigma around cannabis use because of decriminalisation may also result in earlier access to care. It may result in patients being younger at first admission. One can argue, however, that, if patients access care sooner, they may have less severe illness and be more likely to be managed as outpatients, making this unlikely to be the reason for the younger age of admission post-decriminalisation.^32,48^ It is beyond the scope of this study to ascertain the impact of cannabis use on the age of onset and duration of untreated MI. However, the younger age of admission in MHCUs post-decriminalisation remains a concern as a young age of admission may have adverse consequences like disruption in education and work, faster disease progression and increased risk of comorbid metabolic syndrome.^26,52^

Cannabis decriminalisation may worsen outcomes for people living with MI and increase the burden on the healthcare system.

This study suggests that further research on the impact of cannabis decriminalisation is needed, focussing on patient perceptions and risk awareness. Targeted interventions addressing cannabis use are necessary, particularly for young male adults, as this population remained the most admitted post-decriminalisation. Interventions may include youth education, MI screening in young cannabis users and legislation limiting access to cannabis for the youth.^53^ The effectiveness of these interventions requires further research; however, if cannabis use impacts MI prevalence and worsens outcomes in patients with MI, there remains a need for interventions to reduce use. This is especially true at an early age, where there may be implications for the developing brain.^26^

Most patients were unemployed (*n* = 213), and only 23 had a tertiary education (Table 1). Cannabis use and MI are associated with unemployment and poor academic achievement.^2,3^ There was no association between cannabis decriminalisation and level of education or employment status (Table 1). The lack of association between pre- and post-decriminalisation with cannabis use and level of education may be because unemployment and a low level of education were high in the study overall.

### Clinical characteristics

Most patients presented with psychotic symptoms, followed by mood and psychotic symptoms. Mood symptoms in the absence of psychosis were less frequent. Only 12 were intoxicated (Table 2). The higher prevalence of psychotic symptoms among admitted patients could be explained by the severity of mental illness (MI) in this group. Psychotic patients often exhibit impaired insight and judgement, making them less likely to seek treatment voluntarily and more likely to require hospitalisation for supervised care.^25^ They may be at higher risk to themselves and others because of impaired reality testing compared to those who have mood symptoms in the absence of psychosis.^54^ Intoxication is managed in the emergency department, not warranting admission to psychiatry unless there are other comorbid psychiatric concerns.^55^ In keeping with international literature from Canada and America, there was no difference in symptoms pre- and post-decriminalisation.^52^

A third of admissions were first presentations to psychiatry. There was no significant difference pre- and post-decriminalisation in the number of patients admitted with their first episode of MI (Table 3). This study looked at only a small sample of patients from a specific setting and, therefore, cannot comment on changes in the prevalence or incidence of MI post-decriminalisation. However, the decriminalisation of cannabis did not increase the number of first-episode patients being admitted. There may still have been a change in the number of patients presenting with the first episode of MI post-decriminalisation; however, symptom severity may not have warranted admission. This may suggest that decriminalisation has not increased the burden on the healthcare setting by increasing the number of patients being admitted with first-episode psychosis. This study did not assess total admissions or admission duration over time. These are crucial for understanding decriminalisation’s impact on healthcare burden, including new onset MI and relapse rates.

Most patients (*n* = 133) were brought in by family or admitted from MEU (*n* = 162) (Table 2). More patients were brought into the hospital by their families post- than pre-decriminalisation. There was a decrease in the number of patients being brought in by EMS post- compared to pre-decriminalisation. This is in keeping with the literature where most patients (84.2%) were brought to the hospital by family and friends.^56^ Families chose to bring patients to the hospital themselves because of the lack of trust in the EMS in South Africa.^57^ The country has experienced a shortage of EMSs, leading to long waiting times and delayed emergency response.^58^ Cultural beliefs and fear of stigma around MI also influence this decision.^59^ Cannabis use is associated with an increased risk of aggression, and the lack of a significant difference in the number of patients requiring police assistance with transportation to hospital post- versus pre-decriminalisation (*n* = 18 vs. *n* = 7) suggests that decriminalisation has not changed cooperation during admission in patients living with MI.^60^

### Limitations

The retrospective study design posed limitations on the accuracy and completeness of the data, as it depended on pre-existing medical records, which may not always have been thorough or consistently documented. The study specifically examined MHCUs admitted to the CHBAH psychiatry department, and there is a lack of generalisability in the results for MHCUs in the broader South African setting. The anticipated sample size of 256 was not obtained because of missing files and incomplete data, and 44.3% of UMDS results were missing. The small sample size of patients with recorded UMDS results limited statistical power.

Patients were younger post- than pre-decriminalisation; however, the age of index presentation to psychiatry and the age of first-onset cannabis use were not recorded. It was not possible to discern if patients presenting post-decriminalisation were younger because they were using cannabis younger, had earlier onset of symptoms or were accessing care earlier.

It was not possible to comment on diagnosis as this study captured only whether a patient presented with intoxication, psychotic or mood symptoms rather than a specific Diagnostic and Statistical Manual of Mental Disorders (DSM) diagnosis.

Not enough time may have passed since decriminalisation legislation was implemented for its full effects to be felt. Some variables, such as unemployment and poor level of education, were highly prevalent, and the limited sample size of this study may not have been adequate to assess any changes in these demographic characteristics and cannabis use after decriminalisation.

For patients who tested positive for substances such as benzodiazepines and tricyclic antidepressants, we were unable to distinguish whether these substances were medically prescribed or used illicitly, making it difficult to determine the true extent of non-prescribed benzodiazepine use.

Another limitation was the inability to obtain detailed information on the frequency, dosage or specific types of cannabis products used by patients. This gap restricts our understanding of how different patterns of cannabis use may affect mental health outcomes.

Patients were counted only once, preventing analysis of cannabis’s effect on relapse rates. Additionally, the total number of cannabis-related admissions, a key health indicator, was not captured.

## Conclusion

There was a trend towards increased cannabis use post-decriminalisation. Patients post-decriminalisation were younger, suggesting a potential earlier age of use. This study also supports recent findings of an increase in admission post-decriminalisation in adolescent cannabis users in Cape Town.^32^ This is concerning, as decriminalisation in South Africa may lead to increased use among younger patients, which heightens their risk of developing MI at an earlier age. This increased risk is concerning because of its association with poorer treatment outcomes. Evidence suggests that, as cannabis use among youth increases, the likelihood of admission to psychiatric facilities may also escalate, highlighting the need for strategies to mitigate these potential consequences.^26,52,61^

Decriminalisation did not increase self-disclosure of cannabis use significantly, and the clinical benefit of administering UMDS remains unclear. While decriminalisation did not significantly increase overall self-reported cannabis use, there was a statistically significant rise in cannabis use detected via UMDS post-decriminalisation. This suggests that objective testing may reveal increases in cannabis use that self-reporting does not fully capture. Our results also indicate that most MHCUs using cannabis are young males. Being young and male is a risk factor for cannabis use and developing MI, and this study highlights that young males still constitute the majority of admissions post-decriminalisation. Reducing psychiatric admission in young males may lessen the burden on the healthcare system.^24,28,31,39^ The post-decriminalisation period may have fostered a perception that cannabis is safer, contributing to earlier and increased use facilitated by easier access to various cannabis products. If cannabis use in young males has increased, targeted interventions aimed at reducing it in this group may reduce admissions and thus improve individual outcomes and reduce the burden on the healthcare system.^48^
